# Predictors of Postoperative Morbidity in Hepatic Hemangioma Surgery: A Single-Center Experience

**DOI:** 10.3390/medicina62091645

**Published:** 2026-08-27

**Authors:** Ioana Teodora Ciortan (Popa), Catalin-Bogdan Satala, Robert Daniel Ciortan, Irina Balescu, Emanuela Sevastian, Raul Mihailov, Traian Dumitrascu, Nicolae Bacalbasa, Aurel Nechita, Irinel Popescu

**Affiliations:** 1 Clinical and Pharmaceutical Research Center, Faculty of Medicine and Pharmacy, “Dunarea de Jos” University of Galati, 800008 Galati, Romania; ioana.popa@ugal.ro (I.T.C.); irina.balescu@ugal.ro (I.B.); emanuela.sevastian@ugal.ro (E.S.); raul.mihailov@ugal.ro (R.M.); aurel.nechita@ugal.ro (A.N.); irinel.popescu@ugal.ro (I.P.); 2 Fundeni Clinical Institute, Department of Oncological and HBP Surgery, “Carol Davila” University of Medicine and Pharmacy, 050474 Bucharest, Romania; traian.dumitrascu@umfcd.ro (T.D.); nicolae.bacalbasa@umfcd.ro (N.B.); 3 Clinical Emergency County Hospital, 810325 Braila, Romania

**Keywords:** hepatic hemangioma, liver resection, postoperative morbidity, risk stratification, ASA Physical Status Classification, Charlson Comorbidity Index

## Abstract

*Background and Objectives*: Hepatic hemangioma is the most common benign liver tumor and is usually managed conservatively. When surgical treatment is indicated, accurate preoperative risk assessment is important because surgery is elective and aims to relieve symptoms while minimizing postoperative morbidity. This study aimed to identify preoperative predictors of postoperative morbidity following hepatic hemangioma resection. *Materials and Methods:* This retrospective cohort study included 274 consecutive patients who underwent elective hepatic resection for hepatic hemangioma at a tertiary hepatobiliary referral center between January 2002 and December 2024. Demographic, clinical, and operative variables were analyzed. Independent predictors of postoperative morbidity were identified using multivariable binary logistic regression. Model discrimination was assessed by receiver operating characteristic curve analysis, and internal validation was performed using bootstrap resampling. *Results:* Persistent abdominal pain was the most frequent indication for surgery (77.0%) and was significantly more common with increasing tumor size (*p* < 0.001). Maximum tumor diameter (OR 1.329, 95% CI 1.188–1.486; *p* < 0.001), ASA Physical Status Classification (OR 1.791, 95% CI 1.167–2.750; *p* = 0.008), and Charlson Comorbidity Index (OR 1.342, 95% CI 1.015–1.774; *p* = 0.039) were independently associated with postoperative morbidity. Age, sex, and operative approach were not independently associated with postoperative morbidity. The model demonstrated satisfactory calibration (Hosmer–Lemeshow *p* = 0.245) and good apparent discrimination (AUC 0.783, 95% CI 0.722–0.845; *p* < 0.001). The optimism-corrected AUC after internal bootstrap validation was approximately 0.732. *Conclusions:* Tumor size, ASA Physical Status Classification, and Charlson Comorbidity Index independently predicted postoperative morbidity after hepatic hemangioma resection. Their integration may provide a practical preoperative risk-stratification framework for patients being considered for elective hepatic hemangioma surgery. However, external validation in independent cohorts is required before broader clinical application.

## 1. Introduction

Hepatic hemangiomas (HHs) are the most common benign tumors of the liver, with a reported prevalence ranging from 0.4% to 20%, depending on the population studied and the diagnostic method employed. Their widespread detection over recent decades largely reflects the increasing use of abdominal ultrasonography, computed tomography, and magnetic resonance imaging, which have made incidental focal liver lesions a frequent finding in routine clinical practice. Despite being common, HH generally follows a benign natural course. Most remain asymptomatic throughout life, exhibit little or no growth on serial imaging, and rarely require any form of intervention beyond periodic surveillance. This favorable natural history explains why conservative management remains the standard of care for most patients [1,2,3]. Current clinical guidelines recommend surgery only in carefully selected situations, including persistent symptoms attributable to the lesion, progressive tumor enlargement, compression of adjacent organs, spontaneous rupture, Kasabach–Merritt syndrome, or persistent diagnostic uncertainty despite comprehensive imaging assessment. Nevertheless, defining the optimal indication for surgery remains challenging. Symptoms are often nonspecific, lesion size alone does not necessarily correlate with clinical presentation, and the risk of intervention must be balanced against the fact that these tumors are benign. Consequently, the decision to operate is frequently individualized and continues to vary among hepatobiliary centers [1,2,4,5].

The surgical management of HH has evolved considerably during recent decades, with improvements in liver transection, bleeding control, anaesthesia, perioperative care, and the increasing use of laparoscopic surgery [6,7]. Despite these advances, postoperative morbidity remains clinically important because HH is benign and surgery is elective. Reliable preoperative risk assessment is therefore essential for patient selection, perioperative planning, and counselling regarding the risks and benefits of surgery [6,8,9,10].

Several factors have been associated with postoperative outcomes after liver resection. Tumor size, anatomical location, the extent of liver resection, operative blood loss, and surgical complexity have all been reported to influence perioperative morbidity. In patients with HH, larger lesions often present additional technical challenges, including more demanding liver mobilization, distortion of vascular anatomy, and an increased likelihood of major hepatectomy or prolonged operative time. Consequently, previous studies have primarily focused on technical aspects of surgery or on comparisons between enucleation and formal hepatectomy, as well as between laparoscopic and open approaches. Much less attention has been directed toward characteristics of the patient undergoing surgery [6,8,11,12,13].

In routine clinical practice, patient age, physiological reserve, and comorbidities are also considered when major liver resection is planned. ASA Physical Status Classification and the Charlson Comorbidity Index (CCI) are widely used measures of operative risk and chronic disease burden and have been associated with postoperative outcomes in abdominal and hepatobiliary surgery [14,15,16,17]. Whether they provide similar prognostic value in patients undergoing HH resection remains unclear. In addition, the apparent benefits of laparoscopic surgery may be influenced by differences in patient and tumor selection [7,8,18,19,20]. Identifying independent preoperative predictors may therefore provide a more objective framework for risk assessment than comparisons based solely on operative technique.

Based on these aspects, the present study aimed to evaluate preoperative factors associated with postoperative morbidity in a consecutive cohort of patients undergoing HH resection. We specifically investigated whether age, ASA physical status, Charlson Comorbidity Index, tumor size, and operative approach independently predicted postoperative complications. Secondary objectives included exploring the association between tumor size and the clinical indications leading to surgical treatment.

## 2. Materials and Methods

### 2.1. Study Design and Patient Population

This retrospective observational cohort study included 274 consecutive patients who underwent elective hepatic resection for hepatic hemangioma at the Department of General Surgery, Fundeni Clinical Institute, Bucharest, Romania, between January 2002 and December 2024. Patients were identified through the institutional surgical database, and demographic, clinical, operative, and postoperative data were retrieved from electronic medical records. The extended study period was primarily determined by the relative rarity of large hepatic hemangiomas requiring surgical treatment and was considered necessary to assemble a sufficiently large consecutive cohort for meaningful statistical analysis. Because surgical techniques, perioperative management, and patient selection may have evolved during this interval, potential temporal heterogeneity was specifically considered in the statistical analysis.

Only patients with a diagnosis of HH established on the basis of characteristic imaging findings and multidisciplinary clinical evaluation were included in the analysis. Patients undergoing surgery for malignant liver tumors, traumatic liver injury, or other benign hepatic lesions were excluded, as were patients without histopathological confirmation of HH. The inclusion of consecutive patients was intended to minimize selection bias and to reflect routine clinical practice at a tertiary hepatobiliary referral center.

### 2.2. Preoperative Assessment and Surgical Management

All patients underwent a standardized preoperative evaluation including clinical examination, laboratory investigations, abdominal ultrasonography, and contrast-enhanced computed tomography and/or magnetic resonance imaging. The following preoperative variables were collected: age, sex, maximum radiological tumor diameter, ASA Physical Status Classification, Charlson Comorbidity Index (CCI), indication for surgery, and planned operative approach. Tumor size was defined as the largest lesion diameter measured on preoperative cross-sectional imaging. The indication for surgery was established after multidisciplinary hepatobiliary evaluation. Surgical treatment was performed for one or more of the following indications: persistent abdominal pain considered attributable to the hemangioma, progressive tumor enlargement, psychological distress related to the presence of a large hepatic lesion, or persistent diagnostic uncertainty despite comprehensive radiological assessment. For statistical analyses, the principal indication leading to surgery was recorded for each patient. The choice between open and laparoscopic liver resection was based on tumor characteristics, anatomical location, technical feasibility, and surgeon preference. Depending on lesion size and location, surgical procedures ranged from enucleation and limited anatomical liver resections to major hepatectomy. Liver transection techniques and methods of vascular control were selected according to intraoperative findings and surgeon preference. Postoperative morbidity was defined as the occurrence of any complication following HH surgery during the postoperative period. Complications were graded according to the Clavien–Dindo classification, with grades I–II considered minor complications and grades III–V considered major complications. Major morbidity was therefore defined a priori as a Clavien–Dindo grade ≥III event.

### 2.3. Statistical Analysis

Continuous variables were assessed for distribution and are presented as median and range because most variables showed a non-normal distribution. Categorical variables are reported as absolute frequencies and percentages. Comparisons between categorical variables were performed using Pearson’s chi-square test or Fisher’s exact test when appropriate. Differences between continuous variables were evaluated using the Mann–Whitney U test for two-group comparisons and the Kruskal–Wallis test for comparisons involving more than two groups. Associations between ordinal or continuous variables were assessed using Spearman’s rank correlation coefficient. Factors associated with postoperative complications were evaluated using multivariable binary logistic regression. In addition, patients with and without postoperative morbidity were compared using univariable analyses to further characterize the clinical profile associated with postoperative complications. Variables were selected for inclusion in the primary model based on their clinical relevance, previous evidence from the literature, and their availability before surgery rather than solely on univariable statistical significance. The final primary model included age, sex, maximum tumor diameter, ASA Physical Status Classification, Charlson Comorbidity Index, and operative approach. Operative time, estimated blood loss, perioperative transfusion, and other intraoperative parameters were not included in the primary model because they are determined during or after surgery and were not considered preoperative predictors. Tumor location and extent of hepatectomy were not included; these variables were recorded inconsistently throughout the retrospective study cohort. To assess the potential influence of temporal heterogeneity over the extended study period, calendar year of surgery was additionally considered in a sensitivity analysis. The multivariable logistic regression model was therefore repeated after adjustment for year of surgery, while retaining the variables included in the primary model. In addition, a sensitivity analysis was performed by stratifying the cohort into two temporal periods (2002–2012 and 2013–2024) to determine whether the association between maximum tumor diameter and postoperative morbidity remained consistent across different eras of surgical management. The primary multivariable model was retained as the prespecified preoperative model, whereas these additional analyses were performed to assess the robustness of the observed associations to potential temporal changes in surgical practice, perioperative management, and patient selection. As an additional sensitivity analysis, type of surgery (enucleation versus liver resection) was incorporated into the multivariable model to assess whether the association between maximum tumor diameter and postoperative morbidity remained independent of the principal operative strategy. Results are presented as odds ratios (ORs) with 95% confidence intervals (95% CIs). Model calibration was assessed using the Hosmer–Lemeshow goodness-of-fit test, whereas model discrimination was evaluated by receiver operating characteristic (ROC) curve analysis with calculation of the area under the curve (AUC). Internal validation was additionally assessed using bootstrap resampling to estimate optimism in model discrimination and derive an optimism-corrected AUC. All statistical analyses were performed using IBM SPSS Statistics version 26.0 (IBM Corp., Armonk, NY, USA). A two-sided *p* value < 0.05 was considered statistically significant.

## 3. Results

### 3.1. Patient Characteristics

A total of 274 consecutive patients who underwent elective hepatic resection for hepatic hemangioma were included in the study. The baseline demographic, clinical, and operative characteristics of the study population are summarized in Table 1.

The study population was predominantly female, with women accounting for 209 patients (76.3%), while 65 patients (23.7%) were men. The median age at the time of surgery was 49 years (range, 20–75 years), reflecting the predominance of middle-aged patients undergoing surgical treatment for HH.

Most patients presented with relatively large lesions. Hemangiomas measuring 10–15 cm represented the largest subgroup (78.8%), whereas tumors smaller than 10 cm and larger than 15 cm accounted for 10.9% and 10.3% of cases, respectively. These findings are consistent with current surgical practice, where operative treatment is generally reserved for selected patients with large or symptomatic lesions.

Regarding baseline physical status, more than half of the cohort was classified as ASA I, while approximately one-third of patients had an ASA II score and only a minority were classified as ASA III. Similarly, Charlson Comorbidity Index (CCI) scores indicated a relatively low burden of chronic comorbidity, with over two-thirds of patients presenting a CCI of 0 or 1 before surgery.

Open liver resection remained the predominant operative approach, being performed in 213 patients (77.7%), whereas laparoscopic resection was undertaken in 61 patients (22.3%). Overall postoperative morbidity occurred in 75 patients (27.4%). According to the Clavien–Dindo classification, minor complications (grades I–II) accounted for 58 patients (21.2%), whereas major postoperative morbidity (grade ≥ III) occurred in 17 patients (6.2%), including five grade IIIa, four grade IIIb, seven grade IV, and one grade V event. Given the relatively small number of major complications, these events were evaluated descriptively, and no separate multivariable prediction model for major morbidity was constructed because the limited number of events would not support a statistically robust model. One postoperative death (grade V) occurred during the study period. The distribution of postoperative complications according to Clavien–Dindo severity grade is presented in Table 2. The median postoperative length of hospital stay was 9 days (range, 3–25 days). These findings indicate that hepatic resection for hemangioma was associated with an acceptable overall morbidity profile, with major postoperative complications occurring in a minority of patients.

Overall, the study cohort represents a relatively homogeneous population of patients undergoing elective hepatic resection for predominantly large HH, with generally favorable baseline physical status and a low burden of pre-existing comorbidity.

### 3.2. Association Between Tumor Size and Surgical Indications

The distribution of the primary surgical indications according to maximum tumor diameter is summarized in Table 3.

Persistent abdominal pain was by far the most frequent indication for hepatic resection, accounting for 211 patients (77.0%). Psychological distress, progressive tumor enlargement, and persistent diagnostic uncertainty represented substantially less common indications, accounting for 23 (8.4%), 22 (8.0%), and 18 (6.6%) patients, respectively. The distribution of surgical indications differed significantly across tumor size categories (Pearson χ^2^, *p* < 0.001). Notably, all patients with hemangiomas larger than 15 cm underwent surgery because of persistent abdominal pain, whereas the remaining indications were observed exclusively among patients with tumors measuring less than 15 cm. Analysis of tumor diameter as a continuous variable confirmed the same pattern. Tumor size differed significantly among the four indication groups (Kruskal–Wallis H = 18.182, df = 3, *p* < 0.001), with patients undergoing surgery because of persistent abdominal pain presenting the largest lesions (Table 4).

Pairwise post hoc analysis demonstrated significantly larger tumors in this group than in patients undergoing surgery because of progressive tumor enlargement (*p* = 0.003). No statistically significant differences were identified between the remaining indication groups after adjustment for multiple comparisons.

### 3.3. Predictors of Postoperative Morbidity

**Figure 1:**
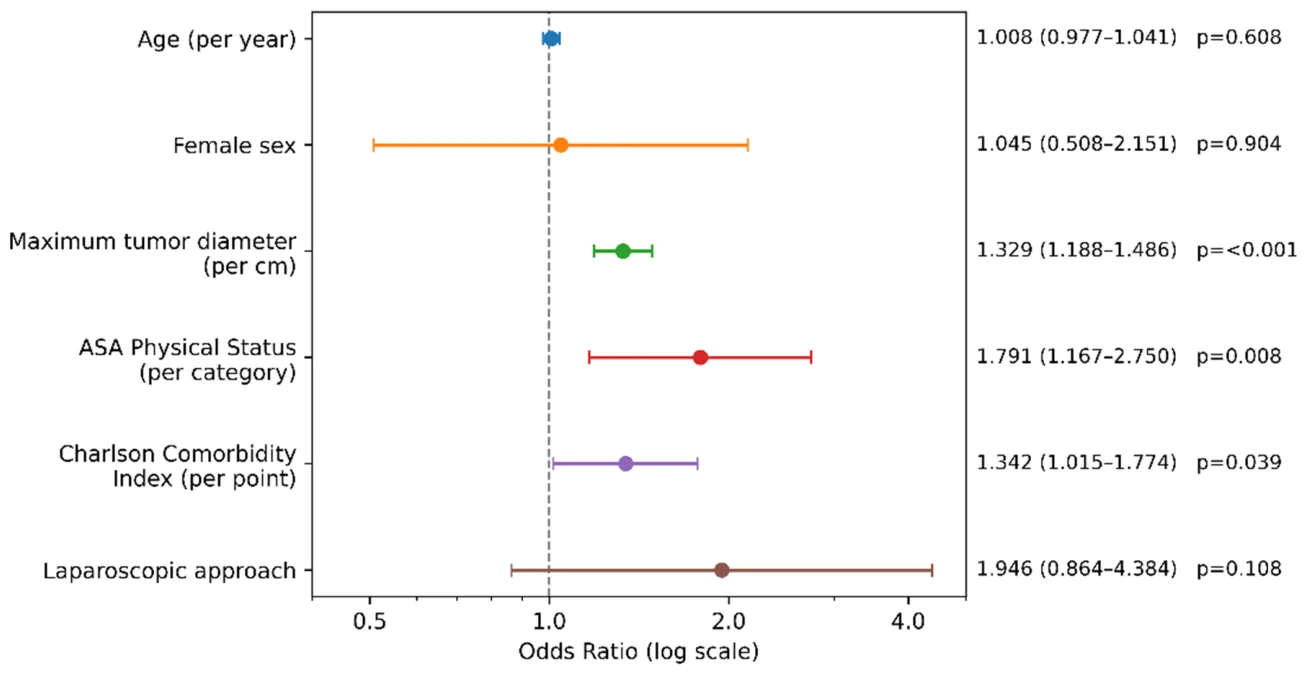
Independent predictors of postoperative morbidity after hepatic hemangioma resection.

To identify independent predictors of postoperative morbidity, a multivariable binary logistic regression model was constructed including age, sex, maximum tumor diameter, ASA Physical Status Classification, Charlson Comorbidity Index, and operative approach. The results of the analysis are summarized in Table 5 and illustrated in Figure 1.

The overall regression model was statistically significant (χ^2^ = 57.579, *p* < 0.001), indicating that the selected variables significantly improved prediction of postoperative morbidity compared with the intercept-only model. Maximum tumor diameter emerged as the strongest independent predictor of postoperative morbidity. Each additional centimeter in tumor diameter was associated with a 32.9% increase in the odds of developing postoperative complications (OR 1.329, 95% CI 1.188–1.486, *p* < 0.001). Higher preoperative ASA Physical Status Classification was likewise independently associated with postoperative morbidity. Each increase in one ASA category was associated with a 79.1% increase in the odds of postoperative complications (OR 1.791, 95% CI 1.167–2.750, *p* = 0.008). Charlson Comorbidity Index also remained an independent predictor. For every one-point increase in CCI, the odds of postoperative morbidity increased by 34.2% (OR 1.342, 95% CI 1.015–1.774, *p* = 0.039). In contrast, neither patient age, sex, nor operative approach independently predicted postoperative morbidity after adjustment for the remaining covariates.

To assess whether the observed associations were influenced by temporal changes during the extended study period, the multivariable model was additionally adjusted for calendar year of surgery. After this adjustment, maximum tumor diameter remained independently associated with postoperative morbidity (OR 1.345, 95% CI 1.196–1.514, *p* < 0.001), with an effect estimate comparable to that observed in the primary model. Calendar year was independently associated with postoperative morbidity (OR 0.895, 95% CI 0.836–0.958, *p* = 0.001). A further sensitivity analysis stratified patients into two temporal cohorts (2002–2012 and 2013–2024). Maximum tumor diameter remained independently associated with postoperative morbidity in both periods, with an OR of 1.379 (95% CI 1.165–1.631, *p* < 0.001) in the 2002–2012 cohort and 1.284 (95% CI 1.102–1.495, *p* = 0.001) in the 2013–2024 cohort. There was no evidence of a significant interaction between tumor diameter and study period (*p* = 0.678), supporting the consistency of this association across the two temporal eras. As an additional sensitivity analysis, type of surgery was incorporated into the multivariable model. After adjustment for enucleation versus formal liver resection, maximum tumor diameter remained independently associated with postoperative morbidity (OR 1.298, 95% CI 1.161–1.452, *p* < 0.001), whereas type of surgery itself was not independently associated with postoperative morbidity (OR 0.825, 95% CI 0.449–1.516, *p* = 0.536).

### 3.4. Clinical Characteristics of Patients Developing Postoperative Morbidity

To provide a more comprehensive clinical characterization of patients experiencing postoperative morbidity, perioperative variables were compared between patients with and without postoperative complications (Table 6).

Although these comparisons were performed at the univariable level, they provide valuable insight into the overall clinical profile associated with an adverse postoperative course. Patients who developed postoperative complications tended to be older than those with an uncomplicated recovery (median age 52 vs. 48 years, *p* = 0.012), suggesting that increasing age may contribute to a reduced physiological reserve following hepatic surgery. In parallel, the morbidity group presented with significantly larger hemangiomas (median diameter 13 vs. 11 cm, *p* < 0.001), a finding consistent with the greater technical complexity generally encountered during resection of bulky lesions. The overall burden of pre-existing medical conditions also differed between groups. While the distribution of ASA classes was comparable (*p* = 0.357), patients who experienced postoperative morbidity demonstrated significantly higher Charlson Comorbidity Scores (*p* = 0.018), indicating that cumulative comorbidity rather than preoperative anesthetic classification alone may better reflect susceptibility to postoperative adverse events. These findings further support the importance of comprehensive preoperative patient assessment in addition to conventional surgical risk stratification.

Differences were also observed in the operative approach. Laparoscopic surgery was performed significantly more frequently among patients with an uncomplicated postoperative course (26.1% vs. 13.3%, *p* = 0.036), whereas the proportion of patients undergoing enucleation or formal liver resection was similar between groups (*p* = 0.982). Although these descriptive comparisons should not be interpreted as evidence of an independent protective effect of the laparoscopic approach, they likely reflect the careful selection of patients with more favorable disease characteristics for minimally invasive surgery. Perioperative blood transfusion was markedly more frequent among patients who subsequently developed postoperative complications (16.0% vs. 2.5%, *p* < 0.001). Rather than representing an isolated perioperative event, transfusion may be viewed as a surrogate marker of increased operative complexity, greater intraoperative blood loss, or technically demanding procedures, all of which are factors commonly associated with postoperative morbidity. As expected, postoperative complications translated into a longer recovery period. Patients in the morbidity group required a significantly prolonged hospital stay compared with those without complications (median 10 vs. 8 days, *p* = 0.001), reflecting the additional postoperative care and clinical monitoring required in this subset of patients.

Overall, this comparative analysis highlights that patients experiencing postoperative morbidity exhibited a distinct perioperative profile characterized by older age, larger tumor size, a greater burden of comorbidities, increased transfusion requirements, less frequent use of minimally invasive surgery, and prolonged hospitalization. These observations complement the multivariable logistic regression analysis by providing a clinically meaningful comparison between the two patient groups and further contextualize the factors associated with postoperative outcomes following hepatic hemangioma surgery.

### 3.5. Predictive Accuracy of the Postoperative Morbidity Model

**Figure 2:**
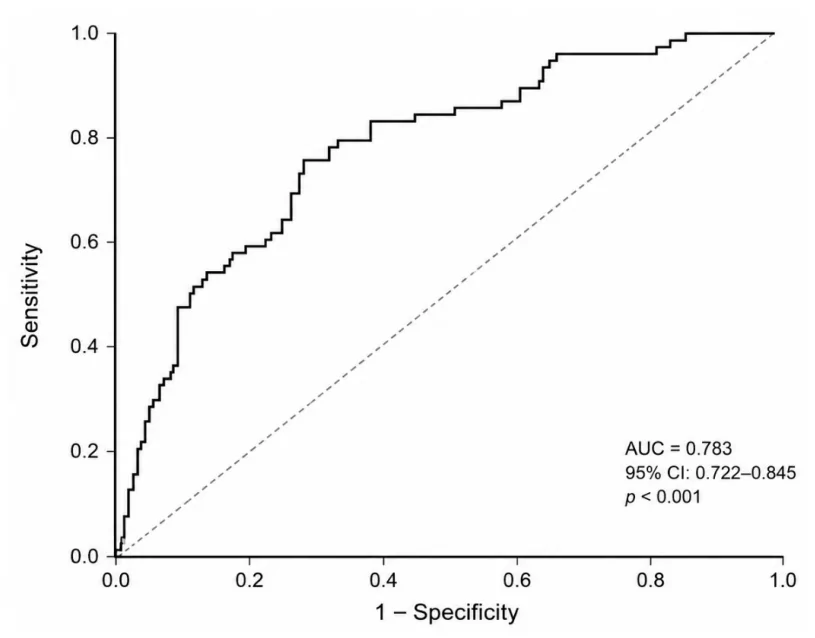
Predictive accuracy of the multivariable model for postoperative morbidity.

Following identification of the independent predictors of postoperative morbidity, the predictive performance of the multivariable logistic regression model was further evaluated. The model demonstrated satisfactory calibration, with good agreement between the predicted and observed postoperative outcomes, as indicated by a non-significant Hosmer–Lemeshow goodness-of-fit test (χ^2^ = 10.290, *p* = 0.245). Receiver operating characteristic (ROC) curve analysis demonstrated good discriminative performance, with an area under the curve (AUC) of 0.783 (95% CI 0.722–0.845, *p* < 0.001), indicating good discrimination between patients who developed postoperative morbidity and those who experienced an uncomplicated postoperative course (Figure 2). The optimal predicted-probability cutoff for the multivariable model was 0.238, corresponding to a sensitivity of 74.7% and a specificity of 66.3% for predicting postoperative morbidity. In an additional internal validation performed on the available study dataset, the optimism-corrected AUC was approximately 0.732, indicating that the model retained acceptable discriminatory performance after correction for potential optimism.

Overall, the model demonstrated good predictive accuracy for postoperative morbidity in patients undergoing hepatic resection for hepatic hemangioma.

## 4. Discussion

Although hepatic resection for hepatic hemangioma (HH) has become considerably safer over the past two decades, determining which patients are most likely to benefit from surgery remains a challenging aspect of hepatobiliary practice. Because surgery is performed for a benign disease and is almost invariably elective, the anticipated symptomatic benefit must always be balanced against the risk of postoperative complications. Identifying patients at increased perioperative risk is therefore essential not only for patient selection, but also for appropriate surgical planning and preoperative counselling [1,6,11,4].

The present study evaluated preoperative factors associated with postoperative morbidity in a large consecutive cohort of patients undergoing HH resection. Persistent abdominal pain was the predominant indication for surgery and became progressively more frequent with increasing tumor size. In addition, maximum tumor diameter, ASA Physical Status Classification, and Charlson Comorbidity Index emerged as independent predictors of postoperative morbidity, whereas operative approach did not retain statistical significance after adjustment for baseline patient and tumor characteristics. Collectively, these findings suggest that postoperative outcomes are determined by the interplay between tumor burden and baseline patient condition rather than by the surgical approach alone [12,16,17]. The main contribution of the present study is not the identification of previously unknown individual risk factors, but their integration into a preoperative risk framework specifically developed for patients undergoing hepatic resection for hepatic hemangioma. The simultaneous assessment of tumor size, ASA Physical Status Classification, Charlson Comorbidity Index, and operative approach allowed the independent contribution of tumor burden and baseline patient condition to postoperative morbidity to be evaluated within the same model. In addition, the association between tumor size and postoperative morbidity remained consistent after adjustment for calendar year, across the two study periods, and after accounting for the principal operative strategy. These findings may therefore provide a more integrated approach to preoperative risk assessment in patients being considered for surgical treatment of HH.

Persistent abdominal pain represented the principal indication for surgery in the present cohort, accounting for more than three quarters of all resections. Moreover, the proportion of patients undergoing surgery because of pain increased progressively with tumor size, with persistent abdominal pain representing the exclusive indication among patients with lesions larger than 15 cm. At first sight, this observation may appear inconsistent with current international recommendations, which emphasize that tumor size alone should not be considered an indication for hepatic resection. However, these findings are not contradictory. Current EASL Clinical Practice Guidelines recommend surgical treatment only for carefully selected patients with persistent symptoms, progressive tumor enlargement, compression of adjacent organs, consumptive coagulopathy, spontaneous rupture, or unresolved diagnostic uncertainty, regardless of lesion size. Our results suggest that increasing tumor diameter does not directly determine the decision to operate but instead increases the likelihood of symptom development, making abdominal pain the principal driver of surgical referral [1,2,21].

This interpretation is supported by previous surgical series. In one of the largest multicenter studies addressing HH surgery, Miura et al. reported that abdominal pain was the indication for resection in the great majority of patients, whereas progressive enlargement and diagnostic uncertainty accounted for only a small proportion of surgical cases. Similar conclusions were reached by Hoekstra et al., who emphasized that symptom burden rather than lesion diameter should guide surgical decision-making. Taken together, these observations reinforce the current concept that tumor size should not be viewed as an isolated indication for surgery but rather as a factor that increases the probability of developing symptoms requiring operative treatment [11,21].

The relationship between tumor size and postoperative morbidity represents a separate issue. In the present study, maximum tumor diameter remained an independent predictor of postoperative complications even after adjustment for patient characteristics and operative approach. This observation is clinically plausible and consistent with surgical experience. Larger hemangiomas frequently require more demanding liver mobilization, more extensive parenchymal transection, and are more often associated with major hepatic resections or technically complex dissections around major vascular structures [6,4]. Previous reports have similarly linked larger hemangiomas to longer operative time, greater intraoperative blood loss, and increased technical complexity [12,22].

While tumor-related factors are central to surgical decision-making, the present findings indicate that baseline patient condition also plays an important role in determining postoperative outcomes. Both ASA Physical Status Classification and Charlson Comorbidity Index remained independently associated with postoperative morbidity after adjustment for tumor size and operative approach, suggesting that the physiological reserve of the patient contributes to postoperative recovery independently of lesion characteristics [15,16,17,23,24].

This observation is in line with the broader hepatobiliary literature. Several studies evaluating patients undergoing liver resection have demonstrated that increasing comorbidity burden and impaired preoperative physical status are associated with higher postoperative morbidity, prolonged hospitalization, and slower recovery. Although these associations have been reported predominantly in cohorts including patients with primary or metastatic liver malignancies, the same principles appear applicable to elective surgery for benign liver disease. Patients undergoing HH resection are often perceived as carrying a relatively low operative risk because they are generally younger and surgery is performed electively. Our results suggest that this assumption should be interpreted with caution, as postoperative outcomes remain influenced by the patient’s overall health status even in this highly selected population [16,17,23,24,25,26].

An additional finding deserves consideration. Although ASA classification and Charlson Comorbidity Index are frequently used together in clinical practice, they evaluate different aspects of preoperative risk. ASA classification reflects the clinician’s assessment of the patient’s overall physiological condition immediately before surgery, whereas the Charlson index provides a standardized estimate of chronic disease burden. The fact that both variables remained significant within the same multivariable model suggests that they provide complementary rather than overlapping information. Consequently, incorporating both measures into routine preoperative assessment may improve risk stratification beyond the use of either score alone [14,15,17,27].

These findings have practical implications for perioperative management. Because HH resection is almost always performed electively, optimization of chronic medical conditions before surgery may be particularly valuable in patients with impaired physiological reserve or a higher burden of comorbidity. Careful assessment of baseline patient fitness therefore appears to be as important as evaluating tumor characteristics when planning surgical treatment [16,28,29].

The role of operative approach in HH surgery has been extensively investigated during the last decade. Several retrospective comparative studies and subsequent meta-analyses have reported lower intraoperative blood loss, reduced postoperative pain, shorter hospital stay, and faster recovery following laparoscopic liver resection compared with conventional open surgery. These observations have contributed to the increasing adoption of minimally invasive liver surgery for benign liver tumors whenever technically feasible. However, interpretation of these data remains challenging because the choice of operative approach is strongly influenced by patient selection. Lesion size, anatomical location, proximity to major vascular structures, anticipated extent of resection, and surgeon experience all contribute to the decision between laparoscopic and open surgery, making direct comparisons inherently susceptible to selection bias [8,30,31].

This issue is reflected in the present study. Although laparoscopic resection was associated with favorable baseline characteristics, operative approach did not remain independently associated with postoperative morbidity after adjustment for tumor size, ASA Physical Status Classification, and Charlson Comorbidity Index. Rather than conflicting with the existing literature, this finding suggests that part of the perioperative advantage observed in minimally invasive series may be attributable to differences in patient and tumor characteristics rather than to the surgical approach itself. Similar concerns have been acknowledged in previous comparative studies, where authors emphasized that laparoscopic liver resection is generally offered to carefully selected patients with technically favorable lesions [7,8,32].

From a clinical perspective, these findings should be interpreted with caution. They do not imply that laparoscopic and open liver resection provide equivalent perioperative outcomes in every setting, nor do they diminish the established role of minimally invasive liver surgery in experienced hepatobiliary centers. Instead, they emphasize that operative approach represents only one component of a broader decision-making process. The present data suggest that baseline patient condition and tumor burden exert a greater influence on postoperative morbidity than the choice of surgical access itself. Consequently, careful patient selection remains the cornerstone of achieving favorable outcomes, irrespective of whether resection is performed through an open or laparoscopic approach [7,8].

**Figure 3:**
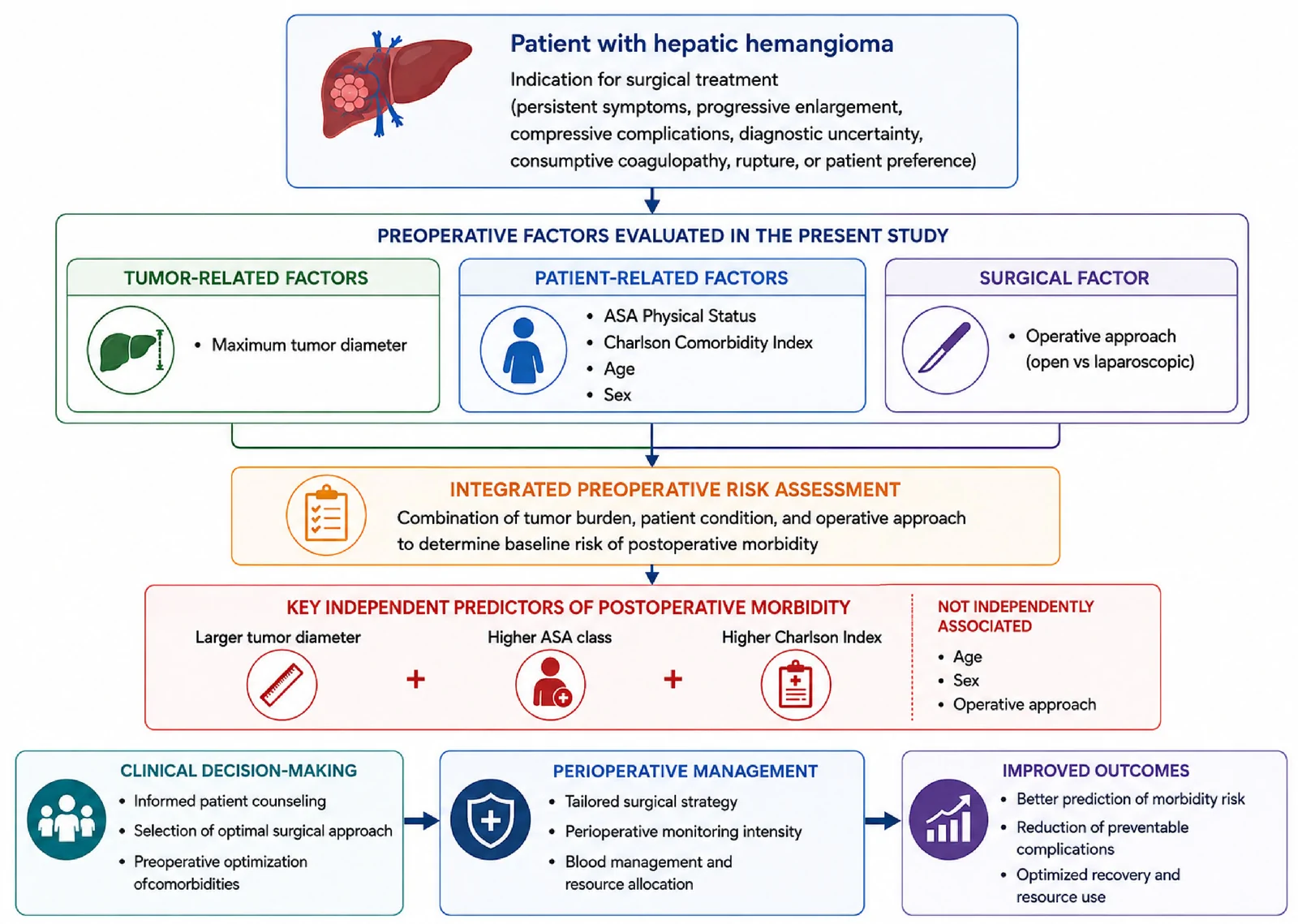
Proposed conceptual framework illustrating the integration of patient-related, tumor-related, and surgical factors into preoperative risk assessment for hepatic hemangioma surgery. The framework summarizes the principal findings of the present study and highlights the potential role of tumor size, ASA Physical Status Classification, and Charlson Comorbidity Index in supporting individualized perioperative decision-making.

The findings of the present study have several practical implications for the preoperative management of patients with HH. Because surgical treatment is elective in almost all cases, the decision to operate extends beyond determining whether resection is technically feasible. Instead, surgeons must balance the expected improvement in symptoms against the potential risk of postoperative morbidity. The present results suggest that this assessment should not rely on a single variable. Rather, tumor burden, baseline physiological status, and comorbidity should all be considered when estimating operative risk and discussing treatment options with the patient [1,6,28]. The conceptual framework proposed in Figure 3 summarizes the principal findings of the present study and illustrates how patient-related characteristics, tumor burden, and surgical considerations can be integrated into a structured preoperative assessment.

This framework is not intended to represent a validated prediction algorithm; it provides a practical representation of the relationships identified in the present analysis and may facilitate clinical decision-making during multidisciplinary evaluation. Future multicenter studies with external validation could determine whether this conceptual model can be refined into a standardized preoperative risk assessment tool for patients undergoing HH surgery. An important observation emerging from this study is that tumor size appears to influence two distinct stages of clinical decision-making. Before surgery, increasing lesion size is associated with a higher probability of symptom development, thereby increasing the likelihood of surgical referral. Once resection is undertaken, however, tumor size also contributes to postoperative risk by increasing operative complexity. At the same time, baseline patient condition independently modifies postoperative outcomes, indicating that technically successful surgery alone does not eliminate the influence of underlying comorbidities. Considering these factors together may therefore provide a more realistic estimate of perioperative risk than evaluating each variable in isolation [10,16].

Although the prediction model demonstrated good discrimination in the present cohort, the additional internal resampling analysis indicated some optimism in the apparent model performance. The model should therefore be regarded as an exploratory preoperative risk-stratification framework rather than a clinically validated prediction tool. Decisions regarding HH resection remain inherently individualized and should continue to incorporate symptom severity, anatomical considerations, surgeon experience, and patient preference. Future multicenter studies incorporating external validation will be valuable to determine whether the present findings can be translated into broadly applicable preoperative risk assessment strategies [33].

One of the strengths of the present study is the inclusion of a relatively large consecutive cohort of patients undergoing hepatic resection for HH over a prolonged study period. Given the rarity of surgical treatment for this benign liver tumor, large single-center experiences remain valuable for understanding factors that influence postoperative outcomes. Another strength is the simultaneous evaluation of tumor-related characteristics and baseline patient condition within the same multivariable model, allowing the independent contribution of each preoperative variable to postoperative morbidity to be assessed. The additional sensitivity analyses further examined the robustness of the observed association between tumor size and postoperative morbidity in relation to temporal changes and operative strategy.

The results should nevertheless be interpreted in light of several limitations. First, the retrospective design inevitably introduces the possibility of selection bias and residual confounding despite multivariable adjustment. Second, this was a single-center study conducted at a high-volume hepatobiliary referral center, and the observed outcomes may therefore not be directly generalizable to institutions with different patient populations or surgical expertise. Because individual postoperative complications were not consistently coded by clinical type throughout the retrospective study period, postoperative morbidity was analyzed according to overall occurrence and Clavien–Dindo severity rather than by complication-specific categories. Although the proposed prediction model demonstrated good discrimination in the present cohort, the additional internal resampling analysis indicated some optimism in the apparent model performance. External validation was beyond the scope of the present study and remains necessary before broader clinical implementation can be recommended. Finally, the long study period may have introduced temporal heterogeneity related to advances in surgical techniques, perioperative care, and patient selection. This potential source of bias was specifically addressed through adjustment for calendar year and sensitivity analyses stratified by study period, which showed that the association between tumor size and postoperative morbidity remained consistent across the two temporal eras. Nevertheless, residual effects of changes in surgical practice and patient selection over time cannot be completely excluded.

## 5. Conclusions

Tumor size, ASA Physical Status Classification, and Charlson Comorbidity Index are independently associated with postoperative morbidity after hepatic resection for hepatic hemangioma. Larger tumor size, higher ASA classification, and greater comorbidity burden identify patients at increased risk of postoperative complications. The association between tumor size and postoperative morbidity remained robust after adjustment for calendar year, across different study periods, and after accounting for the principal operative strategy. The proposed multivariable model demonstrated good discrimination in the present cohort, although internal resampling indicated some optimism in its apparent performance. The model should therefore be considered a preliminary framework for preoperative risk stratification rather than a clinically validated prediction tool, and external validation in independent cohorts is required before routine clinical implementation.

## Figures and Tables

**Table 1:** Baseline demographic and clinical characteristics of the study cohort.

Variable	Overall (*n* = 274)
Age, years	49 (20–75) *
Sex	
Female	209 (76.3)
Male	65 (23.7)
Maximum tumor diameter	
<10 cm	30 (10.9)
10–15 cm	216 (78.8)
>15 cm	28 (10.3)
ASA Physical Status	
ASA I	141 (51.5)
ASA II	104 (38.0)
ASA III	29 (10.6)
Charlson Comorbidity Index	
0	104 (38.0)
1	88 (32.1)
2	44 (16.1)
≥3	38 (13.9)
Operative approach	
Open resection	213 (77.7)
Laparoscopic resection	61 (22.3)
Postoperative morbidity	75 (27.4)
Postoperative length of stay, days	9 (3–25) *

* Data are presented as median (range). All other values are presented as *n* (%).

**Table 2:** Early postoperative outcomes following hepatic hemangioma resection.

Variable	Overall (*n* = 274)
Overall postoperative morbidity	75 (27.4)
Clavien–Dindo grade	
Grade I	37 (13.5)
Grade II	21 (7.7)
Grade IIIa	5 (1.8)
Grade IIIb	4 (1.5)
Grade IV	7 (2.6)
Grade V	1 (0.4)
Major complications (Clavien–Dindo grade ≥ III)	17 (6.2)
Minor complications (Clavien–Dindo grade I–II)	58 (21.2)
Median postoperative length of stay, days *	9 (3–25)

* Data are presented as median (range). All other values are presented as *n* (%).

**Table 3:** Primary surgical indications according to tumor size. * *p* statistically significant if < 0.05.

Primary Indication for Surgery	<10 cm (*n* = 30)	10–15 cm (*n* = 216)	>15 cm (*n* = 28)	Overall (*N* = 274)	*p* Value *
Persistent abdominal pain	21 (70.0)	162 (75.0)	28 (100.0)	211 (77.0)	
Psychological distress	5 (16.7)	18 (8.3)	0	23 (8.4)	
Progressive tumor enlargement (>2 cm/year)	2 (6.7)	20 (9.3)	0	22 (8.0)	
Diagnostic uncertainty	2 (6.7)	16 (7.4)	0	18 (6.6)	
Overall comparison					<0.001

Pearson χ^2^ test. Data are presented as *n* (%).

**Table 4:** Comparison of maximum tumor diameter according to the primary indication for surgery.

Primary Indication for Surgery	*n*	Median Tumor Diameter (cm)	Range (cm)	Mean Rank
Persistent abdominal pain	211	12.8	5.0–22.5	148.40
Psychological distress	23	10.4	6.5–14.8	108.15
Progressive tumor enlargement	22	9.6	5.8–14.2	95.86
Diagnostic uncertainty	18	9.9	6.0–13.7	98.08
Kruskal–Wallis test				H = 18.182
Overall *p* value				<0.001

**Table 5:** Independent predictors of postoperative morbidity.

Variable	Odds Ratio (OR)	95% Confidence Interval	*p* Value
Age (per year)	1.008	0.977–1.041	0.608
Female sex	1.045	0.508–2.151	0.904
Maximum tumor diameter (per cm)	1.329	1.188–1.486	<0.001
ASA Physical Status (per category)	1.791	1.167–2.750	0.008
Charlson Comorbidity Index (per point)	1.342	1.015–1.774	0.039
Laparoscopic approach	1.946	0.864–4.384	0.108

**Table 6:** Comparison of patients with and without postoperative morbidity.

Variable	No Morbidity (*n* = 199)	Postoperative Morbidity (*n* = 75)	*p* Value
Laparoscopic approach, *n* (%)	52 (26.1%)	10 (13.3%)	0.036
Type of surgery, *n* (%)			0.982
• Enucleation	95 (47.7%)	35 (46.7%)	
• Liver resection	104 (52.3%)	40 (53.3%)	
Perioperative blood transfusion, *n* (%)	5 (2.5%)	12 (16.0%)	<0.001
Postoperative length of stay, days, median	8	10	0.001

## Data Availability

The data supporting the findings of this study are available from the corresponding author or first author upon reasonable request.
